# Can People Guess What Happened to Others from Their Reactions?

**DOI:** 10.1371/journal.pone.0049859

**Published:** 2012-11-30

**Authors:** Dhanya Pillai, Elizabeth Sheppard, Peter Mitchell

**Affiliations:** School of Psychology, University of Nottingham, Malaysia Campus, Selangor, Malaysia; University College London, United Kingdom

## Abstract

Are we able to infer what happened to a person from a brief sample of his/her behaviour? It has been proposed that mentalising skills can be used to retrodict as well as predict behaviour, that is, to determine what mental states of a target have *already* occurred. The current study aimed to develop a paradigm to explore these processes, which takes into account the intricacies of real-life situations in which reasoning about mental states, as embodied in behaviour, may be utilised. A novel task was devised which involved observing subtle and naturalistic reactions of others in order to determine the event that had previously taken place. Thirty-five participants viewed videos of real individuals reacting to the researcher behaving in one of four possible ways, and were asked to judge which of the four ‘scenarios’ they thought the individual was responding to. Their eye movements were recorded to establish the visual strategies used. Participants were able to deduce successfully from a small sample of behaviour which scenario had previously occurred. Surprisingly, looking at the eye region was associated with poorer identification of the scenarios, and eye movement strategy varied depending on the event experienced by the person in the video. This suggests people flexibly deploy their attention using a retrodictive mindreading process to infer events.

## Introduction

When two people are engaged in social interaction, they each react to the behaviour of the other, and these reactions could manifest as humour, irritation, sympathy, bashfulness, to name a few. Can we guess what provoked a reaction just by observing a person's behaviour? If so, this might qualify as an instance of what Gallese and Goldman [1] called ‘retrodiction’, which is a kind of backwards inference from a mental state to its causal antecedent. In this case, the mental state is embodied in a reaction (humour, irritation, sympathy, bashfulness, etc). Can participants guess, for example, what caused a person to manifest irritation? If so, then participants would effectively have access to an aspect of the world through the lens of another person's mind (as embodied in behaviour). Indeed, the participants could perhaps learn something about a third party, by observing the effect the third party had on another person. This would be an important faculty in that participants could use other minds as a way of broadening their apprehension of the world – in this particular case, the social world. Apparently, this would qualify as a significant benefit of the participants' capacity for mentalising, or imputing mental states.

Currently, not much research uses tasks that have presented participants with a sample of behaviour and asked them to infer or to ‘retrodict’ the situation that resulted in that behaviour (although see Robinson & Mitchell [2] for an exception). Another aspect of understanding minds in the real world is that not all people will respond to the same situation in the same way (the diversity problem). One-to-one correspondence between situation and behaviour in real life is uncommon and we might assume that the mental states that mediate between situation and behaviour will also vary. Laboratory tasks that involve behavioural prediction tend to artificially generate one-to-one correspondence between situation and behaviour, ignoring this important feature. Paradigms are required that instead take account of this variability in responses with a view to discovering how we flexibly understand the behaviour of others, even where it departs from how we would be inclined to act ourselves.

Some researchers have circumnavigated these issues by presenting participants with samples of behaviour (usually facial expressions) and asking them to identify the mental state of the individual concerned [3], usually without any further inference to the antecedent situation. Proponents of this approach have argued that mental states (such as admire, thoughtfulness, scheme) can be directly observable from facial expressions. They also argue that in our everyday lives we understand the mental states of others through a combination of high-level and low-level mentalising processes. High-level processes involve reasoning in a “top-down” fashion about mental states based on our prior knowledge of the relationships between mental states and situations. For example, based on our prior knowledge about the relationship between what a person sees and what they know, we might reason that someone has a false belief about an object being in a particular location because that individual did not witness it being moved elsewhere. In contrast, low-level mentalising processes involve “bottom-up” recognition of cues or indicators such as eye gaze behaviour or facial expressions [3]. The mechanisms for these two processes may well be different: mirror neurons have been proposed as a possible basis for low-level mentalising processes, while high-level mentalising is more likely to be grounded in a propositional (non-bodily) format [4]. Retrodictive mindreading as described above might involve a combination of these processes. We may well recognise and decode the behaviour via a bodily format of representation, a relatively low-level process. However, this must be at some point integrated with some prior knowledge of situations that may give rise to that kind of feeling.

The stimuli used in studies involving identifying mental states from facial expressions have also been criticised for the following reasons. In real life, the behaviour we are trying to understand may be subtle. For example, facial expressions are often dynamic and brief. Most studies have not taken these considerations into account and have tended to portray static images of emotional responses, often displayed for several seconds [5]. As the expressions in such studies are posed by actors, the “correct” answer as to what that person is thinking or feeling is usually determined by consensus of viewers and may bear no relation to the actual mental state of the actor in question. As no event or situation in the world has given rise to the expression (other than the actor being asked to pose by the researcher), it is unclear how well results of such studies can inform us about the processes we use when reasoning about the relationship between an event or situation and observable behaviour.

In recognition of this last point some researchers have developed more naturalistic stimuli where expressions have either been induced or recorded in a real-world setting. For example, Matsumoto, Olide, Schug, Willingham and Callan [6] used facial expressions of athletes, captured at the end of Olympic Judo matches. While we can be more confident that the individuals reacting in studies such as this are experiencing some kind of mental state, a problem remains in knowing the correct answer. Even if the individual him/herself is asked what he/she was thinking or feeling we cannot know for certain whether the verbal report is an accurate representation of the mental state experienced. Besides, the individual may feel a blend of emotions, thoughts, desires and so on, many of which cannot easily be described in words. It may also be that it is easier to perceive or interpret the behaviour of an individual than to generate a mental state label that adequately captures an impression of his or her experience. Moreover, the very act of trying to verbalise what another person might be thinking or feeling could interfere with our ability to spontaneously interpret their behaviour in context.

Concerns about difficulties associated with naming mental states (in this case, belief states) were taken into consideration by Wimmer and Perner [7] when they devised the now widely known unexpected transfer test of false belief, in which child participants are invited to predict where a protagonist will search for chocolate (and are not asked to make any direct inferences about the protagonist's belief state). In this task, participants must reflect on the belief state of the protagonist in order to predict his/her behaviour, but are not required to name or refer to any belief state directly. While that research undoubtedly represented a major breakthrough, it is open to the criticisms mentioned previously (i.e. one-to-one correspondence between situation and behaviour).

The research reported in this paper aimed to develop a paradigm that approximates many of the demands of real life situations where mental state reasoning might be required, and to address some of the criticisms that might be levelled against previous research. Participants were shown people's natural (and somewhat subtle) reactions to four specific events (which we will refer to as scenarios), all of which were filmed during an interaction with the researcher, and asked to identify which of the four events had previously occurred. To succeed at this task, it is necessary to retrodict, that is, to reason backwards from behaviour to infer a situation that had already happened. Participants were not asked to identify the mental state of the individuals explicitly, thus overcoming any concerns about their being able to verbalise or label their inner subjective states and avoiding the possibility of our “instructing” participants to use a mentalising strategy. Instead, participants were required to identify the situation, about which there was a definite objectively correct answer. If a small sample of behaviour is sufficient for people to get a feel for what circumstance may have led to that behaviour, then we predict that participants would systematically identify which of the four specific events had previously occurred to the people in the videos that they viewed.

We also recorded participants' eye movements while viewing the videos. Previous research has suggested that when viewing static images containing people, individuals look more at the eye region compared to the rest of the face [8]. Also, when freely viewing videos containing people, both adults [9] and young children [10] tend to spend the majority of their time looking at the eye region of the face. Moreover, the eye region may convey crucial information for tasks that involve trying to name emotions or mental states from images of faces [3]. Given the importance of the eye region in previous research, we predicted that participants would spend more of their time looking at the eye region than the mouth region of the faces of the people in the videos. We also hypothesised that time spent looking at the eye region would correlate with successful identification of the event that had previously occurred. In other words, we predicted that participants who spent more time looking at the eye region when viewing the videos would have the greatest success at identifying the scenarios. We also hypothesised that this relationship would hold for each of the scenarios i.e. we predicted that time spent looking at the eye region when viewing a particular scenario would positively correlate with identification of that scenario.

## Methods

The entire procedure was approved by the Ethics Committee, School of Psychology, University of Nottingham.

### Stimulus development

The purpose of this stage of the study was to create stimuli to be used in the main experiment. Participants were told that they would be filmed while posing some facial expressions to act as stimuli for another study. Unknown to the participants, the real aim was to record their responses to an aspect of the researcher's behaviour that occurred prior to recording the posed expressions. More details are provided below.

#### Participants

Forty participants (19 males and 21 females) aged between 19 and 34 (mean age = 22.2 years) from University of Nottingham were filmed reacting to an apparently incidental aspect of the researcher's behaviour. Participants were of various nationalities: 16 Malaysians, 12 British, 1 Spanish, 1 Vietnamese, 1 Sri Lankan, 1 Botswanan, 1 Indian, 1 Italian, 1 Irish, 1 Nigerian, 1 Polish, 1 Chinese, 1 Ugandan, and 1 Lithuanian. Written informed consent was obtained from all participants.

#### Materials and apparatus

A spacious room within the School of Psychology was utilised. Participants sat with their back towards the main door and windows (so as to avoid distractions). The researcher sat across the table from the participant.

A Sony DCR-TRV460 video camera was used to film participants. The camera was positioned approximately 1.7 meters from the participant and was placed directly next to the researcher on a tripod. The camera was positioned in order for the participant's face, neck, shoulders, and chest to be seen.

#### Procedure

Participants were told that they would be filmed while posing specific facial expressions which would be used as video stimuli in a subsequent study. Four scenarios were created, one of which was performed by the researcher to each participant. The scenarios were devised with a view to eliciting a range of responses from participants. We aimed to create events that would provoke a reaction but would be unlikely to cause a major disturbance in the mood of the participant. Scenarios also needed to be plausible within the context of an experiment, as it was important that participants did not guess that the researcher was acting.

Scenario 1 (Joke): As the participant was ready and waiting to start the experiment, the researcher initiated a short casual chat with him/her. The researcher then shared a simple joke with the participant. The joke was:

*“Why did the woman wear a helmet at the dinner table? Because she was on a crash diet!”*

Scenario 2 (Waiting): As the participant was ready and waiting to start the experiment, the researcher kept the participant waiting for about 5–8 minutes while she performed other irrelevant tasks (i.e. making a phone call, texting on a mobile phone, having a drink of water) while sitting in front of the participant.

Scenario 3 (Story): As the participant was waiting to start the experiment, the researcher began to relate a story about a series of misfortunes she experienced earlier that day, such as *missed the bus to university, left mobile phone at home, caught in rain with no umbrella*, and *flashdrive containing important work malfunctions*.

Scenario 4 (Compliments): As the participant was waiting to start the experiment, the researcher gave instructions regarding the experiment. While doing so, the researcher offered a series of compliments. Examples are:

- *Really nice pair of earrings you have there*
- *You've got really good hair, what shampoo do you use?*
- *That shirt really brings out the colour of your eyes!*

As the real aim was to record participants' immediate responses to the four scenarios, the video camera was set to record as soon as participants were seated; participants were unaware the camera was recording at this stage. At the completion of any one of the four scenarios mentioned above, participants were asked to look directly at the video camera and to form six facial expressions (*surprise*, *happy*, *fear*, *anger*, *sad*, *disgust*). The facial expression words were dictated by the researcher in the same order each time. As the participants were unaware that filming had already begun, the researcher pretended to switch on the camera prior to dictating the facial expression words. Once the participants had completed posing the six expressions, the researcher turned off the video camera. Prior to leaving the testing room, participants were debriefed about the true nature of the study and were given the opportunity to ask any questions. Participants' consent to use the video recording of their reactions to the researcher in Scenario 1–4 was obtained. One participant did not provide consent and the related recordings were destroyed immediately (this participant is not included in the 40 stated above).

#### Editing

The footage was transferred from the video camera to an Apple Macintosh computer using video-editing software, iMovie HD 6. The videoclips were edited to capture participants' reactions to the distinct scenarios at points where they were deemed to be most expressive. Due to the naturalistic and temporally distinct context of the scenarios, there was no clear way of determining a definite start and end point to each reaction as every individual responded uniquely to the varying scenarios. This opened up the possibility of experimenter bias in picking the most stereotypical responses as the editor was not blind to the scenarios when viewing and editing the videoclips. Nevertheless, most of the videos captured responses around the end of the scenario enactment. The 40 edited videoclips (10 for each scenario) ranged from 3.64 to 8.96 seconds, based on the dynamic nature of the participants' natural responses with the respective means being Joke: 6.59 (*SD* = .26); Waiting: 6.84 (*SD* = .23); Story: 6.86 (*SD* = .37); Compliments: 5.81 (*SD* = .40). A one-way ANOVA showed that the clip length did not vary systematically with the scenarios (*p* = .116). Video frames were 720 pixels in width and 576 pixels in height. The rate of presentation was 25 frames per second. The edited clips omitted the audio component as participants' verbal responses would have completely disambiguated the reactions in many cases.

### Main Experiment

#### Participants

Thirty-five participants (19 males and 16 females) aged between 18 and 35 (mean age = 22.37 years) took part in this phase of the study. The experiment was conducted at the University of Nottingham. Participants were of various nationalities: 19 Malaysians, 7 British, 3 Sudanese, 1 Dutch, 1 Japanese, 1 Singaporean, 1 Indonesian, 1 Tanzanian, and 1 Chinese national. All were paid an inconvenience allowance and written informed consent was obtained.

#### Materials and apparatus

Videoclips of the researcher acting each of the four scenarios were filmed using a Sony DCR-TRV460 video camera. The researcher looked directly at the camera while acting. These videoclips of the researcher acting out the scenarios were transferred from the video camera to an Apple Macintosh computer using iMovie HD 6 software. They were then edited using VirtualDub (v1.9.10) video capture and video processing software. VirtualDub was used to create coloured borders for each scenario clip: Scenario 1 (Joke)- Green border, Scenario 2 (Waiting)- Red border, Scenario 3 (Story)- Blue border, and Scenario 4 (Compliments)- Yellow border. The coloured borders were 0.5 centimetres in width. Clips varied in length according to the content of the social scene; Scenario 1 (Joke)- 11 seconds, Scenario 2 (Waiting)- 89 seconds, Scenario 3 (Story)- 34 seconds, and Scenario 4 (Compliments)- 27 seconds. These variations in length were inevitable because of the dynamics of the encounters themselves. Video frames were 720 pixels in width and 576 pixels in height. The rate of presentation was at 25 frames per second. The bit rate for the audio track was 352 kbps.

The stimuli for this study were the 40 edited videoclips from the Stimulus Development stage. All videos (both the researcher enacting the scenarios and the participants' reactions) were shown on a 17 inch TFT monitor which was incorporated into the Tobii T60 (data rate 60 Hz). The 40 stimuli were shown using Tobii Studio Analysis Software. The software randomised the presentation of the videoclips. Each videoclip was interspersed with an image of a fixation point (white central cross on a black background located at the centre of the screen). The fixation point remained on the screen until the participant responded to the previously presented videoclip. The Tobii T60 Eye Tracker was used to record participants' looking behaviour. The participants sat approximately 60 centimetres from the monitor. The video stimuli presented subtended a horizontal visual angle of 22.5° and a vertical visual angle of 11.4°.

To correspond with the scenario videoclips with coloured borders, four matching flashcards with the dimensions 10 centimetres×14 centimetres were created with borders of the same colours. The names of each scenario (*Joke*, *Waiting*, *Story*, *Compliments*) were printed in black ink on white background. The coloured borders on the flashcards were approximately 0.5 centimetres in width and were used to aid memory for the scenarios as acted by the researcher. Only the scenario clips and flashcards had corresponding coloured borders to aid memory recall. The 40 edited videoclips did not have coloured borders. The coloured borders in the scenario clips and flashcards could have influenced participants to respond a certain way, e.g. to pick the waiting scenario more often because they liked the colour red. However, this would result in a higher false alarm rate, which is taken into account by d-prime calculation.

#### Design

A within-subjects design was used, where all participants viewed the four scenario videoclips followed by the 40 reaction expressions clips.

#### Procedure

Participants were tested individually in a quiet room. The videoclips of the researcher enacting the four scenarios were shown on the eye tracker screen. The scenarios were presented in counterbalanced order and were only shown once to participants at the start of the experiment. After the presentation of each scenario videoclip, the corresponding flashcard for the scenario was shown to the participants and placed on the table in front of the monitor. The flashcards were thus displayed in the same counterbalanced order as the scenario videos had been presented. Presenting these scenario clips allowed participants to experience as closely as possible the experience of the participants in the Stimulus Development phase. As a result, participants had a clear understanding of what each scenario entailed, with both audio and visual information presented. Prior to the start of the experiment, a 9-point calibration procedure was conducted in which a moving red dot appeared in different locations on the screen, including the centre, the four corners and the mid-points in between. Following successful calibration, participants were shown the 40 videoclips of reactions resulting from the scenarios in the Stimulus Development phase, also on the Eye tracker monitor. The presentation of the videoclips was randomised via Tobii Studio Analysis Software. Participants were told to direct their gaze at a central fixation point prior to the presentation of each of the 40 videoclips, which was controlled via mouse-click by the researcher. After the clip was shown, the fixation point reappeared and participants were required to state which scenario they thought the person in the video was reacting to by either verbalising or pointing, using the flashcards as cues. The researcher asked a question each time “Which of these events had just happened?” and then briefly reminded the participants of the four options verbally (i.e. in the same counterbalanced sequence the scenario clips and flashcards were previously presented), while pointing to the flashcards: “Is it the joke, waiting, the story, or compliments?” The researcher recorded participants' responses on a data sheet. This process continued for all 40 videoclips.

## Results

Our primary question was whether participants could discriminate between the four scenarios. Responses were analysed using a signal detection procedure to account for any bias in responding with a particular scenario. This generates an index d-prime (*d*′), from the hit rate (the proportion of occasions on which the participant correctly identified the scenario) and false alarm rate (the proportion of occasions on which the participant identified the scenario when it was the incorrect answer). In this experiment, the hit rate was calculated for the ten trials comprising a particular scenario, while the false alarm rate was calculated across the remaining thirty trials which did not comprise that scenario. The Snodgrass and Corwin [11] correction factor was applied to the hit and false alarm rate calculations to correct for cells containing 0, by adding 0.5 to all cells. *d*′ is then calculated by subtracting the *z*-score for the false alarm rate from *z*-score of the hit rate [*d′* = *Z*(hit rate)−*Z*(false alarm rate) where function *Z*(*p*), *p* ∈ [0,1], is the inverse of the cumulative Gaussian distribution. *d*′ is a measure of the distance between signal and noise distributions and is essentially an indicator of how well participants were able to correctly discriminate each scenario from the others. Table 1 displays the mean accuracy rates, false alarm rates and *d*′ scores for the four scenarios. If participants did not systematically discriminate the correct scenarios, the hit rate would be equal to the false alarm rate and their *d*′ score would be 0. *d*′ scores were significantly greater than 0 for all four scenarios [Joke *t*(34) = 12.61, *p*<.0005; Waiting *t*(34) = 20.83, *p*<.0005; Story *t*(34) = 12.87, *p*<.0005; Compliments *t*(34) = 10.51, *p*<.0005], indicating that participants were able to discriminate between them in a systematic manner.

**Table 1:** Participant mean accuracy rates, false alarm rates and d′ scores (correct scenario discriminability).

	Number correct out of 10 (% in brackets)	False alarms out of a possible 30 (% in brackets)	D′ (*d*-prime)
Joke	4.54 (45.4%)	3.69 (12.3%)	1.06
Waiting	9.00 (90.0%)	3.66 (12.2%)	2.49
Story	5.66 (56.6%)	3.86 (12.9%)	1.32
Compliments	5.11 (51.1%)	4.49 (15.0%)	1.05

To establish if there were differences between the scenarios in participants' level of success, a one-way repeated measures ANOVA was conducted on the *d*′ scores, with scenario as the within-participants factor. There was a main effect of scenario, *F*(3,102) = 87.87, *p*<.0005, Cohen's *f* = 2.73 (large effect; [12]). Posthoc *t*-tests with Bonferroni corrected alpha level of .0083 showed this was due to the Waiting scenario being easier to discriminate than the other three scenarios (all *ps*<.0005). The Story scenario also approached significance in being easier to discriminate than the Compliments scenario (*p* = .009).

### Eye tracking (gaze time) analyses

The purpose of the eye tracking analyses was to determine whether participants' ability to discriminate the scenarios correctly was associated with looking at specific parts of the scene. The eye-tracking data were processed using Tobii Studio 3.0's dynamic areas of interest (AOIs) function. This allows one to create AOIs that move and change shape with the movements of objects in the video. In order to calculate eye movement metrics, AOIs were defined separately on the eye and mouth regions of the video stimuli. The Total Fixation Duration (seconds) metric was used to measure the total duration for all fixations within a) the eye region, and b) the mouth region. Fixation is defined by the standard Tobii fixation filter as two or more consecutive samples falling within a 35 pixel radius.

**Figure 1:**
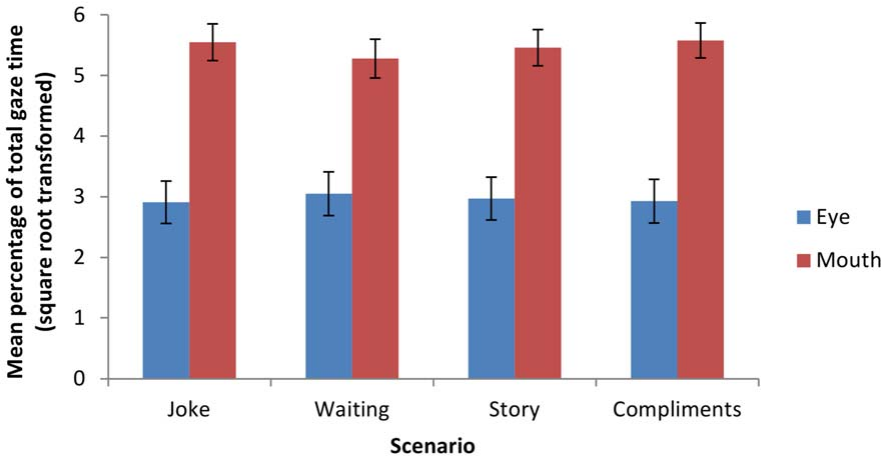
Mean percentage of total gaze time (square root transformed) at the eye and mouth across four scenarios. Error bars report standard errors of the mean.

As the videoclips varied in length, the percentage of gaze time spent on the eyes and mouth regions for each clip was used (i.e. time spent looking at eye/mouth region÷total gaze time * 100%). As the data were not normally distributed (i.e. Shapiro-Wilks tests showed that gaze time at the eye region was not normally distributed for all 4 scenarios, all *ps*<.0005. This was due to mild positive skew with all values >.14), the data were transformed using a square root transformation for the purpose of analysis. Following transformation, the data were normally distributed (Shapiro-Wilks *p*>.1). Figure 1 shows the mean gaze time (after transformation) for the eye and mouth (as a percentage of total gaze time) for each of the four scenarios (please note that the standard errors bars in the figure reflect between-subject variance, and are therefore not suitable for assessing within-subject comparisons). A repeated measures ANOVA was conducted on gaze time, with scenario (Joke, Waiting, Story, Compliments) and region of the face (eyes or mouth) as within-participants factors. We included scenario as a factor due to the possibility that participants would use different viewing strategies for the different scenarios. There was no overall effect for scenario (*p* = .065), However, there was an effect for face region, *F*(1,34) = 17.51, *p*<.0005, Cohen's *f* = .69 (large effect), whereby participants spent longer looking at the mouth region (*M* = 5.47, *SD* = 1.77) than the eye region (*M* = 2.97, *SD* = 2.10). This was qualified by a significant interaction between scenario and face region indicating that gaze time to the critical regions (eyes and mouth) did vary with scenario, *F*(3, 102) = 13.06, *p*<.0005, Cohen's *f* = 1.02 (large effect).

Further analyses were conducted to establish the basis of this interaction. Separate one-way ANOVAs examined the effect of scenario on gaze time at the eyes, and the mouth. There was a significant effect of scenario on time spent looking at the eye region, *F*(3,102) = 5.05, *p* = .018, Greenhouse Geisser corrected, Cohen's *f* = .59 (large effect). Posthoc *t*-tests with a Bonferroni corrected alpha level of .0083 revealed that participants spent more time looking at the eye region in the Waiting scenario compared with the Story (*p* = .001) and Joke scenarios (*p*<.0005). Similarly, there was a significant effect of scenario on time spent looking at the mouth region, *F*(3,102) = 11.46, *p*<.0005, Greenhouse Geisser corrected, Cohen's *f* = .95 (large effect). Posthoc *t*-tests with a Bonferroni corrected alpha level of .0083 revealed that participants spent more time looking at the mouth region in the Joke, Compliments, and Story scenarios compared with the Waiting scenario (all *ps*<.0005).

Do variations in gaze pattern relate to accuracy in detecting the scenario? Due to the Waiting scenario being easier to identify than the other three scenarios and showing different eye gaze patterns, it was analysed separately from the other three scenarios when examining the relationship between gaze patterns and scenario identification. This was to ensure that any apparent relationships were not solely driven by performance in this particular condition. Overall there was a significant negative correlation between mean eye region gaze time and mean *d*′ scores (correct scenario discriminability) across the remaining three scenarios, *r* = −.443, n = 35, *p* = .008, suggesting that individuals who spent more time looking at the eye region in general were less successful at discriminating between the scenarios. The relationship between mean mouth region gaze time and mean *d*′ scores was not significant, *r* = .15, n = 35, *p* = .385; indicating no relationship between looking at the mouth and successfully discriminating between the scenarios. The same relationships were also investigated for each scenario individually. In other words, for each scenario we examined whether gaze time at the eye region for that scenario related to *d*′ scores for the same scenario. Eye region gaze time correlated negatively with *d*′ for the Compliments scenarios (*r* = −.34, n = 35, *p* = .032) and the Story scenario (*r* = .44, n = 35, *p* = .008) but not for the Joke scenario (*r* = .02, n = 35, *p* = .91). The Waiting scenario was investigated separately, revealing a similar pattern whereby eye region gaze time correlated negatively with *d*′ (*r* = −.45, n = 35, *p* = .006). There were no significant relationships between gaze time at the mouth region and *d*′ for any of the four scenarios.

## Discussion

Participants were able to deduce from a relatively brief sample of behaviour which of various situations the individual in question had experienced. This implies that participants utilised successful strategies to retrodict the ‘cause’ of the specified response [1], despite considerable diversity in the manner in which people reacted to each scenario. Thus, from observing just a few seconds of a person's reaction, it appears we can gauge what kind of event might have happened to that individual with considerable success. It is not clear from the current results exactly how long a sample of behaviour needs to be in order to support successful identification, and this may be a question for future research. Nevertheless, this capacity constitutes a powerful tool for learning about events in the world, enabling us to benefit indirectly from the experiences of others.

The Waiting scenario was identified more accurately in comparison to the other scenarios. This could be due the nature of the behavioural responses themselves, as the responses in the Waiting scenario often included not only facial expressions but also gestures that could assist in identifying the scenario, such as yawning, sighing, or looking around. The other three scenarios were identified somewhat less successfully, presumably because the behaviours involved were more similar. For example, laughter was a fairly frequent response for all three scenarios. This is one shortcoming of a forced-choice procedure: success in selecting the correct answer is inevitably influenced by how similar it is to other incorrect options. The scenarios we selected for this study elicited a range of reactions, but their degree of similarity was not easy to anticipate.

An alternative explanation for the better discrimination for the Waiting scenario is that the video of the researcher enacting this scenario was longer than for the other three scenarios, because of the nature of the event itself, which was a period of waiting. It is not immediately obvious that a longer event would necessarily be understood better than a shorter event, but we cannot rule this out as a possibility. Nevertheless, the clips of the behavioural responses themselves did not systematically vary in length with the scenario experienced, so participants could not have used a low-level strategy such as the length of the clip they viewed to discriminate between the scenarios.

Eye movement analyses revealed that participants varied their strategy according to the scenarios. For all four of the scenarios, participants focused primarily on the mouth with less time spent looking at the eyes. Nevertheless, for the Waiting scenario participants spent slightly more time looking at the eyes and less time looking at the mouth than for the other three scenarios, suggesting that the eyes were more informative for this scenario than the others. As discrimination was better in the Waiting scenario and participants looked more at the eyes when viewing this scenario than at the others, one might be tempted to conclude that spending longer looking at the eyes does indeed result in better identification. However, the increased looking at the eyes may have been caused by features of the eye gaze behaviour of the individuals in the videos. The people subjected to the Waiting scenario were not in direct interaction with the researcher, and so were more inclined to look around the scene rather than at the researcher compared to individuals in the other scenarios. These eye movements of the people in the videos may have attracted the attention of the observers viewing them, and made the scenario easy to recognise. Given that the relatively strong discrimination performance in the Waiting scenario and the increased looking to the eyes might have been the result of lower-level strategies such as these, the relationship between gaze behaviour and scenario discrimination was analysed separately for the Waiting scenario and the other three (Joke, Compliments, Story).

Surprisingly, looking at the eye region was associated with poorer identification for the three of the four scenarios and unrelated for the fourth (Joke). These results suggest that the eyes are not the most informative facial region when determining what happened to the people in the videos. They stand in contradiction to some studies which imply that typically developing individuals look more at the eyes than the mouth when viewing videos of other people [9] and appear to dispute previous studies which have claimed that the eyes are crucial for mental state understanding [3]. Instead, they suggest that participants may find different parts of the face informative, depending on the specific situation. This is consistent with Cunningham et al. [13] who reported that the mouth region is central in communicating information about certain mental states. More recently, Kirchner, Hatri, Heekeren and Dziobek [14] reported increased fixation time in the mouth region as compared to the eye region in emotional recognition conditions (i.e. conditions high in social salience).

We have already mentioned that there are a number of differences between the demands of the task reported here and other mentalising tasks that have been reported previously. We have argued that one of the strengths of this paradigm is that participants were never asked to identify the mental state of the individuals in the videos. It is possible that, if we had asked participants to attempt to deduce the mental state, they might have gazed more at the eye region. Alternatively, the preference for the mouth could be a result of the dynamic nature of the videos. Although the people in the videos were not interacting with the participants who viewed them, they were interacting with the researcher at the time of filming, who was sitting next to the camera. This effectively placed the participants who viewed the videos within the interaction, which represents a departure from previous methodologies [9].

Before considering the broader implications of these findings, there is a limitation of the current experiment that should be noted. The use of flashcards as a cue to recalling the various scenarios meant that we were unable to record response times for the task. Although participants were not instructed to respond quickly and were given as much time as they needed to make an accurate decision, response times could potentially have given additional information about how difficult participants found the task. A further difficulty with using flashcards is the possibility that some bias could have arisen from the researcher's involvement in the procedure. However, the order of presentation of the cards was carefully counterbalanced to ensure that cues to the correct response were not provided.

Is it possible that participants could infer what happened to the person in the video without mentalising at all? An argument could be advanced that participants ‘match’ behaviour to situation according to a system of behavioural rules [15]. For example, is it possible that participants associated a smiling face with the Joke scenario? This seems implausible, given the wide range of behavioural responses produced by the people in reaction to the various scenarios: in most cases, there is no simple matching between the scenario and the facial expression.

It has recently been argued that we may perform some mentalising tasks such as recognising emotions through activation of representations that have bodily formats (mirror neurons being a main candidate for this), without generating any higher-level propositional representations of mental states. Goldman and de Vignemont [4] refer to this as ‘low-level mentalising’, but argue that other non-embodied processes might be involved at later stages of emotion recognition, such as at the stage of attributing the emotion itself. Similarly, Gallese [16] acknowledges that emotions can be understood via either an embodied process, or a more explicit propositional route through “cognitive elaboration of their visual properties”. In our task where there was no requirement to identify the mental states of the individuals in the videos at all, a stronger case might be made for the more direct route from observing the behaviour to understanding the situation.

Research with infants has demonstrated that by the age of one, babies may be able to understand intentional goal-directed actions [17] and appear to show sensitivity to the belief state of other individuals before two years of age [18]. While it is surely the case that these abilities are supplemented by more sophisticated and explicit propositional representations of mental states with age, it seems unlikely that these low-level mindreading processes become obsolete. It is plausible to suggest that these processes might be engaged in our task due to its emphasis on making sense of behaviour rather than naming a mental state. Given the changing views on the nature of mentalising processes, it seems ever more important to channel our efforts into devising tasks such as the one reported here that closely approximate how we understand other people's behaviour in real life situations.

In summary, from a brief sample of a few seconds of behaviour, adults are able to infer an event that happened to another individual. This appears to be evidence of a powerful retrodictive mindreading process, which might allow us to benefit indirectly from the experiences of others. Looking at the eyes was not a successful strategy for deducing what had happened to the individual in question, and participants tended to vary their viewing strategy according to what the individual in the video had actually experienced. This suggests that participants are affected by the cues present in the person's behaviour to attend to the parts that will be most informative for making sense of it.
